# Long-term outcome of intrastromal corneal ring segments in keratoconus: Five-year follow up

**DOI:** 10.1038/s41598-018-36668-7

**Published:** 2019-01-22

**Authors:** Min-Ji Kang, Yong-Soo Byun, Young-Sik Yoo, Woong-Joo Whang, Choun-Ki Joo

**Affiliations:** Department of Ophthalmology and Catholic Institute for Visual Science, Seoul St. Mary’s Hospital, College of Medicine, The Catholic University of Korea, Seoul, Republic of Korea

## Abstract

To evaluate the effectiveness of intrastromal corneal ring segment (ICRS) use in keratoconus after five years. ICRS has been widely used to correct astigmatism and improve visual acuity in keratoconus. Although the short-term outcome is well known to be effective, long-term outcome has rarely been reported. A retrospective chart review was done. A total of 30 eyes diagnosed with keratoconus and treated with INTACS (Addition Technology, Sunnyvale, CA, USA) were included. Visual acuity, refraction, indices of corneal irregularity, and higher-order aberration were evaluated at preoperative, two months, one year, three years, and five years postoperatively. Uncorrected distance visual acuity (UDVA) and spherical and spherical equivalent were improved (*p* < 0.05) for three years. However, they worsened (*p* < 0.05) at five years to preoperative values. On the other hand, corrected distance visual acuity (CDVA) was improved for five years (*p* < 0.05). Topographic keratometry was flattened, and corneal irregularity indices were improved at five years (all *p* < 0.05). Coma RMS was improved (*p* < 0.05) continuously for five years. ICRS has advantages in improving CDVA with topographic stabilization and decreasing coma in keratoconus for five years.

## Introduction

Keratoconus is a progressive, bilateral, and non-inflammatory disease. Corneal irregular astigmatism and decrease of vision are induced by corneal thinning and protrusion^1,2^. Various treatment options are available. Spectacles or rigid gas-permeable contact lenses can be tried to improve visual acuity. In patients with contact-lens intolerance, intrastromal corneal ring segments (ICRSs) can be used to reduce corneal astigmatism and myopia by flattening the cornea. These days, corneal collagen crosslinking is widely performed to improve astigmatism and halt progression. For very severe cases, keratoplasty can be one option^3–7^.

ICRSs were first introduced to correct myopia in 1991^8,9^. However, now they are more widely used for keratoconus and ectatic diseases than for myopia^10^. Corneal flattening is induced by arc shortening. Corneal segments act as a spacer in lamella. They can thicken the peripheral cornea and flatten the central cornea. This effect is known to be directly proportional to corneal thickness and inversely proportional to corneal diameter^11,12^.

Three types of ICRS are available: INTACS (Addition Technology, Sunnyvale, CA, USA), Kerarings (Mediphacos, Belo Horizonte, Brazil), and Ferrara rings (Mediphacos, Belo Horizonte, Brazil). INTACS is subdivided into two models: INTACS and INTACS SK. INTACS is 6.77 mm in inner diameter, with a hexagonal cross section. INTACS SK is 6.0 mm in inner diameter. It is more effective than INTACS in severe ectatic cases^13^. It has a round cross section to minimize glare^13^.

Several studies have reported that INTACS is effective in keratoconus^14–20^. Uncorrected distance visual acuity and spherical equivalent can be improved after INTACS implantation^14–20^. However, these reports mainly focused on short-term (less than one year) outcomes. Long-term outcomes of INTACS have been reported in some studies, but there is no consensus yet. Some studies have reported that topographic and refractive outcomes are improved in the long term^21,22^, but another study has reported regression in the long term following short-term improvement^6^.

INTACS can influence higher-order aberrations (HOA) and the quality of vision. However, only a few studies have reported short-term changes of HOA^23,24^. Long-term changes of HOA after INTACS have not been reported yet, to be best of our knowledge. Therefore, the objective of this study was to evaluate refractive and topographic parameters and HOA changes after INTACS implantation with a five-year follow up.

## Patients and Methods

### Inclusion and exclusion criteria

This study was performed by retrospective chart review. Eyes diagnosed as keratoconus that had received INTACS were included. The surgery was performed from January 2010 to December 2012 in Seoul St. Mary’s Hospital. Keratoconus stages 1 to 3, based on the Amsler-Krumeich classification, were included. Patients younger than 35 years at the time of surgery were included. These patients who were followed up for more than five years were included. They were evaluated on the preoperative day and at two months, one year, three years, and five years postoperatively. Eyes with a history of ocular surgery or active ocular disease other than keratoconus were excluded. Any complications, such as ring extrusion, perforation into anterior chamber, and infiltration around the ring, were also excluded. This study was approved by the Institutional Review Board (IRB) of Seoul St. Mary’s Hospital and followed the tenets of the Declaration of Helsinki. Informed consent was obtained from all participants.

### Surgical procedure

For all cases, two INTACS (6.0 mm in inner diameter) were inserted symmetrically. Implantation axis and depth were chosen according to the nomogram defined by the manufacturer using ORB scan II (Bausch & Lomb, Rochester, NY, USA).

All surgeries were performed by a single surgeon (C.K.J.). The corneal tunnel was made with a femtosecond laser (Intralase, Irvine, CA, USA) under topical anesthesia. After the tunnel was made, INTACS were inserted, and the corneal incision was sutured with 10-0 nylon. After the surgery, topical antimicrobial and topical steroid four times a day each were used for one month. The corneal suture was removed at one month postoperatively.

#### Visual and refractive outcome

Uncorrected distance visual acuity (UDVA), corrected distance visual acuity (CDVA), and manifest refraction (sphere and cylinder) were measured. We evaluated mean anterior simulated keratometry (anterior SimK), cylinder of anterior simulated keratometry (anterior SimKcyl), mean anterior keratometry in a 3.0-mm zone (anterior 3 mm K), cylinder of anterior keratometry in a 3.0-mm zone (anterior 3 mm Kcyl), mean posterior simulated keratometry (posterior SimK), and cylinder of posterior simulated keratometry (posterior SimKcyl) using Pentacam (Oculus, Inc., Wetzlar, Germany).

#### Corneal thickness

Corneal thickness was measured using Pentacam, to identify the thinnest corneal thickness (TCT), central corneal thickness (CCT), and mean peripheral corneal thickness (PCT) for the superior, nasal, inferior, and temporal corneal thickness of the 6.0-mm area.

#### Index of corneal irregularity

Indices of corneal irregularity provided by Pentacam^1^ were measured to evaluate changes of corneal irregularity. Index of surface variance (ISV, the irregularity of curvature of the anterior corneal surface), index of vertical asymmetry (IVA, asymmetry of curvature between the superior and inferior cornea), keratoconus index (KI), and minimum radius of curvature (*R*_min_, the smallest radius of curvature of the cornea) were evaluated.

#### Higher-order aberration

Corneal wavefront aberrations at 6.0 mm pupil were evaluated with Pentacam. Spherical aberration (4, 0), root mean square (RMS) value of coma (3, ±1), trefoil (3, ±3), and total corneal higher-order aberrations (HOA) (3rd to 6th order HOA) were recorded.

#### Statistical analysis

Statistical analysis for normality was done using a Kolmogorov-Smirnov test. A paired *t*-test was performed for parametric data, and a Wilcoxon signed-rank test was performed for non-parametric data to compare the preoperative and postoperative values. Repeated-measures analysis of variance (ANOVA) was used to evaluate three or more timepoints. All statistical analyses were performed using SPSS software version 22.0 (IBM Corp., Armonk, NY, USA) and *p* < 0.05 was considered to be statistically significant.

## Results

### Demographics

There were 87 eyes that received INTACS from January 2010 to December 2012. Among them, nine eyes were older than 35 years, 18 had keratoconus stage 4, and four were complicated cases (one ring extrusion, two infiltrations around the ring, and one corneal opacity); these were excluded. Another 26 eyes not followed up at at least one timepoint were also excluded. A total of 30 eyes (16 right and 14 left) of 23 consecutive patients (seven bilateral and 16 unilateral; 14 men and nine women) were included. Their mean age was 27.69 years ± 6.68 (SD) (range, 16 to 35 years). Six (20%), 13 (43.33%), and 11 (36.66%) eyes were at stages 1, 2, and 3, respectively, based on the Amsler-Krumeich classification at the time of INTACS implantation.

### Visual outcome

**Figure 1:**
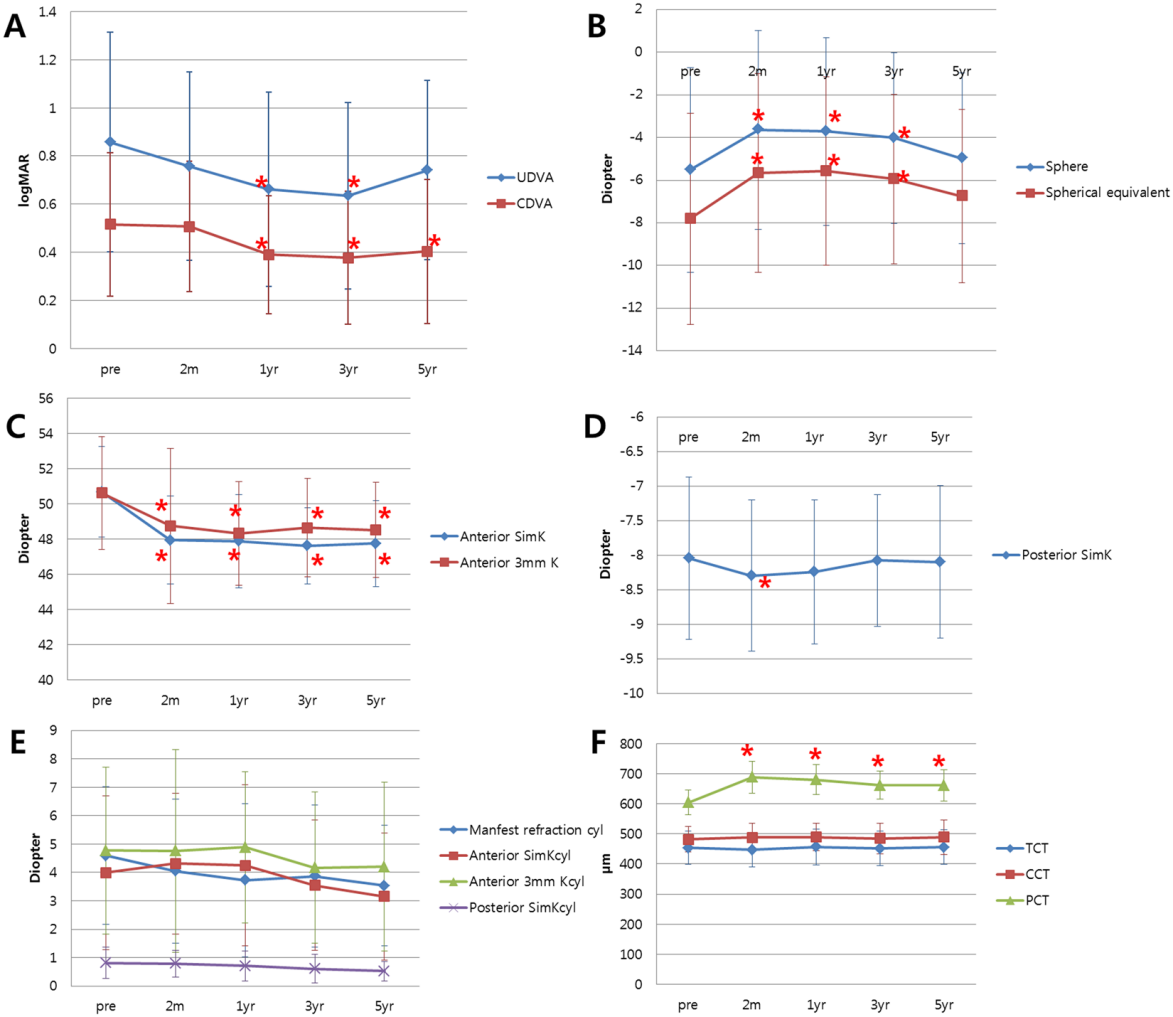
Changes of parameters over time. (**A**) Visual acuity change. (**B**) Sphere and spherical equivalent change. (**C**) Anterior keratometric change. (**D**) Posterior keratometric change. (**E**) Cylinder change. (**F**) Corneal thickness change. UDVA = Uncorrected distance visual acuity. CDVA = Corrected distance visual acuity. Anterior SimK = Mean anterior simulated keratometry. Anterior 3 mm K = Mean anterior keratometry in 3.0-mm zone. Posterior SimK = Mean posterior simulated keratometry. Anterior SimKcyl = Cylinder of anterior simulated keratometry. Anterior 3 mm Kcyl = Cylinder of anterior keratometry in 3.0-mm. Posterior SimKcyl = Cylinder of posterior simulated keratometry. TCT = Thinnest corneal thickness. CCT = Central corneal thickness. PCT = Peripheral corneal thickness. *Indicates statistical significance.

UDVA was improved significantly at one year (*p* = 0.000). Such improvement (*p* = 0.000) was maintained up to three years postoperatively. However, UDVA became worse at five years postoperative, although not worse than the preoperative UDVA (*p* = 0.074) (Fig. 1A and Table 1). CDVA was improved at one year. Such improvement was maintained for five years (*p* = 0.008, *p* = 0.002, and *p* = 0.011 at one year, three years, and five years, respectively) (Fig. 1A and Table 1).

**Table 1:** Visual acuity and refraction before and after INTACS implantation.

Parameter	Preoperative	Postoperative				P value			
		2 m	1 yr	3 yr	5 yr	Pre to 2m	Pre to 1yr	Pre to 3 yr	Pre to five yr
UDVA (log MAR)	0.857 ±0.456	0.757 ±0.391	0.662 ±0.404	0.636 ±0.387	0.741 ±0.372	0.224	0.000*	0.000*	0.074
CDVA (log MAR)	0.516 ±0.298	0.507 ±0.270	0.391 ±0.245	0.377 ±0.276	0.404 ±0.299	0.224	0.008*	0.002*	0.011*
Sphere	−5.521 ±4.791	−3.638 ±4.661	−3.707 ±4.401	−4.021 ±3.996	−4.984 ±4.008	0.000*	0.005*	0.042*	1.000
Spherical equivalent	−7.816 ±4.946	−5.664 ±4.670	−5.571 ±4.424	−5.952 ±3.973	−6.752 ±4.058	0.000*	0.000*	0.001*	0.563

UDVA = Uncorrected distance visual acuity. CDVA = Corrected distance visual acuity.  * indicates statistical significance.

### Refractive outcome

Both sphere and spherical equivalent were improved at two months. Such improvement was maintained up to three years (*p* = 0.000, *p* = 0.005, and *p* = 0.042 at two months, one year, and three years, respectively) (Fig. 1B and Table 1). However, myopia worsened at five years to about its preoperative value (*p* = 1.000).

Anterior SimK was flattened at two months, and this effect was maintained for five years (*p* = 0.001, *p* = 0.000, *p* = 0.001, and *p* = 0.000 at two months, one year, three years, and five years, respectively) (Fig. 1C and Table 2). Anterior 3-mm K in Pentacam was also flattened at two months and maintained for five years (*p = *0.001, *p* = 0.000, *p* = 0.005, and *p* = 0.001 at two months, one year, three years, and five years, respectively) (Fig. 1C and Table 2). Posterior SimK was temporarily steepened at two months. It recovered to the preoperative level at one year (*p* = 0.007 and *p* = 0.139 at two months and one year, respectively) (Fig. 1D). Types of cylinder (manifest refraction cylinder, anterior SimKcyl, anterior 3 mm Kcyl, or posterior SimKcyl) were not changed. They remained constant for five years (Fig. 1E).

**Table 2:** Keratometric change before and after INTACS implantation.

Parameter	Preoperative	Postoperative				P value			
		2 m	1 yr	3 yr	5 yr	Pre to 2 m	Pre to 1 yr	Pre to 3 yr	Pre to five yr
Anterior SimK	50.667 ±2.581	47.921 ±2.497	47.858 ±2.657	47.600 ±2.183	47.733 ±2.434	0.001*	0.000*	0.001*	0.000*
Anterior 3 mm K	50.614 ±3.203	48.728 ±4.410	48.307 ±2.948	48.629 ±2.792	48.500 ±2.715	0.001*	0.000*	0.005*	0.001*
Posterior SimK	−8.047 ±1.176	−8.297 ±1.095	−8.242 ±1.045	−8.075 ±0.952	−8.100 ±1.104	0.007*	0.139	1.000	1.000

Anterior SimK = Mean anterior simulated keratometry; Anterior 3 mm K = Mean anterior keratometry in 3.0 mm zone; Posterior SimK = Mean posterior simulated keratometry.  * indicates statistical significance.

### Corneal thickness

Thinnest corneal thickness (TCT) or central corneal thickness (CCT) was not changed for five years (Fig. 1F). Peripheral corneal thickness (PCT) was increased at two months but slightly decreased at three years (*p* = 0.014). However, PCT was thicker than its preoperative value for five years (*p* = 0.000 at two months, one year, three years, and five years) (Fig. 1F).

### Index of corneal irregularity

ISV showed a decreasing tendency, reaching statistical significance at five years (*p* = 0.030) (Table 3). IVA decreased at three years and five years (*p* = 0.013 and *p* = 0.010, respectively) (Table 3). KI also decreased at three years. This effect was maintained for five years (*p* = 0.039 and *p* = 0.027 at three years and five years, respectively) (Table 3). *R*_min_ was increased at one year and maintained for five years (*p* = 0.001, *p* = 0.001, and *p* = 0.003 at one year, three years, and five years, respectively) (Table 3).

**Table 3:** Corneal irregularity indices before and after INTACS implantation.

Parameter	Preoperative	Postoperative				P value			
		2 m	1 yr	3 yr	5 yr	Pre to 2m	Pre to 1yr	Pre to 3 yr	Pre to five yr
ISV	100.43 ±34.798	104.17 ±32.417	98.36 ±32.993	91.36 ±28.397	89.64 ±28.659	0.366	1.000	0.150	0.030*
IVA	0.951 ±0.515	1.060 ±0.516	1.020 ±0.491	0.888 ±0.451	0.876 ±0.452	0.169	0.744	0.013*	0.010*
KI	1.259 ±0.155	1.265 ±0.143	1.255 ±0.158	1.218 ±0.145	1.215 ±0.140	1.000	1.000	0.039*	0.027*
Rmin	5.637 ±0.512	5.764 ±0.606	5.888 ±0.569	5.971 ±0.480	5.957 ±0.506	0.233	0.001*	0.001*	0.003*

ISV = Index of surface variance; IVA = Index of vertical asymmetry; KI = Keratoconus index; Rmin = Minimum radius of curvature. * indicates statistical significance.

### Higher order aberration

**Figure 2:**
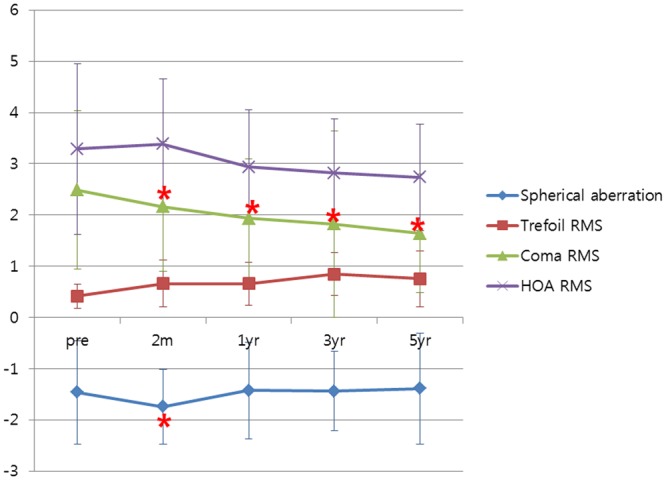
Changes in higher-order aberrations over time. RMS = Root mean square. *Indicates statistical significance.

Spherical aberration was negatively increased at two months. However, it recovered to the preoperative level at one year (*p* = 0.000 and *p* = 1.000 at two months and one year, respectively) (Fig. 2). Coma RMS continuously decreased from two months to five years (*p* = 0.000, *p* = 0.007, *p* = 0.002, and *p* = 0.005 at two months, one year, three years, and five years, respectively) (Fig. 2 and Table 4). Otherwise, trefoil RMS or HOA RMS did not change for five years.

**Table 4:** Higher-order aberrations before and after INTACS implantation.

Parameter	Preoperative	Postoperative				P value			
		2 m	1 yr	3 yr	5 yr	Pre to 2m	Pre to 1yr	Pre to 3 yr	Pre to five yr
Spherical aberration	−1.461 ±1.708	−1.743 ±0.737	−1.425 ±0.944	−1.440 ±0.771	−1.388 ±1.081	0.000*	1.000	1.000	1.000
Trefoil RMS	0.416 ±0.235	0.661 ±0.457	0.656 ±0.421	0.845 ±0.425	0.753 ±0.548	0.267	1.000	0.726	0.141
Coma RMS	2.484 ±1.548	2.161 ±1.269	1.937 ±1.150	1.821 ±1.821	1.642 ±1.163	0.000*	0.007*	0.002*	0.005*
HOA RMS	14.541 ±1.664	15.344 ±1.275	14.365 ±1.112	13.474 ±1.052	12.448 ±1.044	0.357	0.268	0.248	0.071

RMS = Root mean square; HOA = Higher-order aberration.  * indicates statistical significance.

## Discussion

Long-term follow-up study of INTACS is rare. Change over time has rarely been reported. Also, published studies have analyzed only refractive and topographic findings but not higher-order aberrations^6,21,22^, which can influence visual quality after corneal surgery. Its importance has been emphasized these days. Therefore, we looked for changes in refractive, topographic, and aberrational outcomes over time.

UDVA was improved at one year and maintained constant for three years. However, it decreased at five years to preoperative level (Fig. 1A and Table 1), perhaps because of refraction change. After the surgery, sphere and spherical equivalent were improved at two months and maintained for three years. However, myopia was increased at five years close to its preoperative value (Fig. 1B). Increase of myopia at five years can decrease UDVA.

Unlike UDVA, CDVA was improved at one year and maintained for five years (Fig. 1A), perhaps because of topographic regularization. Anterior 3 mm K measured at the corneal center 3.0 mm using Pentacam was flattened at two months and maintained for five years (Fig. 1C). Anterior SimK was also flattened at two months and kept constant for five years (Fig. 1C). Indices of corneal irregularity were also improved. ISV, the index of irregularity of anterior surface curvature, gets higher when the cornea is becoming irregular. In our study, ISV gradually decreased, reaching statistical significance at five years (Table 3). High IVA indicates high vertical asymmetry of curvature. This index also decreased from three to five years (Table 3). KI, representing the severity of keratoconus, also decreased from three to five years (Table 3). *R*_min_, the inverse of corneal steepness, was expected to decrease in keratoconus. However, it was significantly increased from one year to five years (Table 3).

Cylinder has been reported to be decreased after INTACS implantation in many other studies^6,22,23,25^. Only one study has reported that there is no difference from the preoperative cylinder value^16^. Interestingly, no types of cylinders were changed throughout five years in our study (Fig. 1E). Although the cornea was significantly flattened after INTACS implantation (Fig. 1C), the cylinder was not affected at all. Thus, although INTACS can flatten the cornea, it might not have enough power to decrease the cylinder itself.

PCT was increased at two months but slightly decreased at three years, although it was still thicker than it was preoperatively (680.21 µm and 661.69 µm at one year and three years, respectively, *p* = 0.014) (Fig. 1F). Ezra *et al.*^26^ have reported abnormal accumulation of fibrotic extracellular matrix components and proteinases near INTACS, suggesting ongoing lysis and remodeling of corneal stroma. Although PCT was thickened for five years because of the spacer effect of INTACS, the slight decrease of PCT in the current study might be related to remodeling of corneal stroma. Otherwise, TCT or CCT was not changed throughout five years (Fig. 1F).

**Figure 3:**
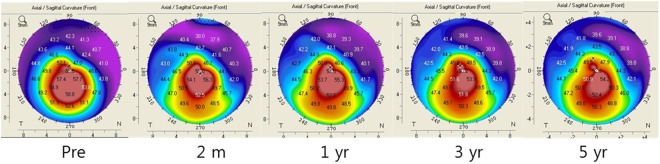
Topographic changes in anterior axial map.

In HOA, only coma RMS continuously decreased throughout five years (Fig. 2), perhaps because of flattening and decreased corneal irregularity (Figs 1C, 3 and Table 3). HOA change after INTACS implantation in keratoconus has rarely been reported. Furthermore, long-term HOA change after INTACS implantation has not been sufficiently evaluated yet. Pinero *et al*. have reported that coma-like aberration is decreased in keratoconus after KeraRing implantation at three months^23^. Coma-like aberration has also been reported to be decreased in post-LASIK ectasia after INTACS implantation at two years^24^. Similarly, coma RMS decreased after surgery, and coma RMS continuously decreased for five years in the present study. Coma has been demonstrated to have a negative effect on visual acuity because of optical blur^27^. Therefore, decrease of coma along with topographic regularization might have contributed to improvement of CDVA in this study.

For the posterior cornea, posterior SimK was temporarily steepened at two months, although it recovered to the preoperative level with a small change (−8.047 to −8.297 from preoperative to postoperative two months, respectively, *p* = 0.007) (Fig. 1D). Posterior SimKcyl was not changed throughout five years (Fig. 1E). INTACSs were implanted into deep corneal stroma. This might have influenced posterior corneal shape. However, in this study, there was no significant change in posterior cornea for five years.

A corneal suture was made after INTACS implantation to prevent ring extrusion or intrastromal infection. This suture can influence the corneal surface. However, a corneal suture was removed after one month, and we analyzed the outcome after two months postoperatively. The effect of the suture on the cornea seemed to be negligible; so we did not take it into account when analyzing outcomes.

The limitation of this study is its small sample size. For this reason, we cannot categorize keratoconus stages from 1 to 4 separately. Given the lack of data, we could not evaluate whether eyes were progressing or not before the surgery. Further studies are needed to overcome these limitations.

In summary, we found that UDVA and refractive outcome were improved for three years. Although spherical and spherical equivalent showed myopic regression, CDVA was improved for five years because of topographic regularization and decrease of coma. Otherwise, there was no thinning of corneal thickness for five years.

In conclusion, ICRS has the advantage of improving CDVA with topographic stabilization. It can improve coma in stages 1 to 3 keratoconus for five years.

## Data Availability

The datasets generated during and/or analyzed during the current study are available from the corresponding author on reasonable request.
